# The antioxidant, anti-inflammatory, and anti-apoptotic effects of sesamin against cisplatin-induced renal and testicular toxicity in rats

**DOI:** 10.1080/0886022X.2024.2378212

**Published:** 2024-07-16

**Authors:** Ahmed E. Altyar, Ghadeer M. Albadrani, Sameh M. Farouk, Mariam K. Alamoudi, Amany A. Sayed, Zuhair M. Mohammedsaleh, Muath Q. Al-Ghadi, Rasha Mohammed Saleem, Hader I. Sakr, Mohamed M. Abdel-Daim

**Affiliations:** a Department of Pharmacy Practice, Faculty of Pharmacy, King Abdulaziz University, Jeddah, Saudi Arabia; b Pharmacy Program, Batterjee Medical College, Jeddah, Saudi Arabia; c Department of Biology, College of Science, Princess Nourah bint Abdulrahman University, Riyadh, Saudi Arabia; d Cytology and Histology Department, Faculty of Veterinary Medicine, Suez Canal University, Ismailia, Egypt; e Department of Pharmacology, College of Pharmacy, Prince Sattam Bin Abdulaziz University, Al-Kharj, Saudi Arabia; f Zoology Department, Faculty of Science, Cairo University, Giza, Egypt; g Department of Medical Laboratory Technology, Faculty of Applied Medical Sciences, University of Tabuk, Tabuk, Saudi Arabia; h Department of Zoology, College of Science, King Saud University, Riyadh, Saudi Arabia; i Department of Laboratory Medicine, Faculty of Applied Medical Sciences, Al-Baha University, Al-Baha, Saudi Arabia; j Department of Medical Physiology, General Medicine Practice Program, Batterjee Medical College, Jeddah, Saudi Arabia; k Department of Medical Physiology, Faculty of Medicine, Cairo University, Giza, Egypt; l Department of Pharmaceutical Sciences, Pharmacy Program, Batterjee Medical College, Jeddah, Saudi Arabia; m Pharmacology Department, Faculty of Veterinary Medicine, Suez Canal University, Ismailia, Egypt

**Keywords:** Cisplatin, cyclooxygenase, inflammation, oxidative stress, p53, sesamin

## Abstract

**Purpose:**

The present study investigated the nephron-testicular protective effects of sesamin against cisplatin (CP)-induced acute renal and testicular injuries.

**Methods:**

Thirty-two male Wistar rats were allocated to receive carboxymethylcellulose (0.5%, as sesamin vehicle), CP (a single i.p. 5 mg/kg dose), CP plus sesamin at 10 or 20 mg/kg orally for 10 days.

**Results:**

Data analysis showed significant increases in serum urea, creatinine, interleukin (IL)-1, IL-6, and tumor necrosis factor-α (TNF-α), as well as renal and testicular tissue malondialdehyde and nitric-oxide concentrations in CP-intoxicated rats in comparison to control animals. On the contrary, rats treated with CP only exhibited significantly lower (*p* < .05) serum testosterone, tissue glutathione, and activities of endogenous antioxidant enzymes compared to control rats. Histopathologically examining CP-intoxicated rats’ tissues using H&E and PAS stains showed atrophied glomeruli, interstitial inflammatory cells, atypic tubular epithelium with focal apoptosis, and reduced mucopolysaccharide content. Further, immunohistochemical staining of the same group revealed an increase in p53 and cyclooxygenase-II (Cox-II) expression in renal and testicular tissues. Treatment with sesamin alleviated almost all the changes mentioned above in a dose-dependent manner, with the 20 mg/kg dose restoring several parameters’ concentrations to normal ranges.

**Conclusions:**

In brief, sesamin could protect the kidneys and testes against CP toxicity through its antioxidant, anti-inflammatory, and anti-apoptotic effects.

## 1. Introduction

Cisplatin (CP) is a platinum-based anti-cancer drug used widely in chemotherapy for treating several cancers, such as those of the testes, lung (non-small cell lung cancer), bladder, head and neck, and cervix [1–3]. Upon cell entry, CP undergoes aquation, and the hydrolyzed product of this process binds to the reactive N7 center in the purine residues. This binding causes significant DNA damage, replication arrest, transcription inhibition, and apoptosis in malignant cells [4]. Despite these benefits, nephrotesticular toxicities remain significant obstacles to the success of CP treatment. Recent reports suggested that 21–31.5% of patients treated with CP develop acute kidney injury (AKI) [5,6]. Other studies showed that CP may cause male infertility and hypogonadism, but the exact involved mechanisms remain unconfirmed [7]. However, CP remains the backbone of many treatment regimens owing to the lack of more efficient alternatives. Thus, searching for compounds that can alleviate its toxic effects is pursued [8].

Biologically active, plant-derived natural compounds have caught attention as potential therapeutics in recent years. Sesamin is a fat-soluble lignan extracted from sesame oil and seeds [9]. Recent reports highlighted several biological benefits of sesamin, including anti-inflammatory, neuroprotective, anti-hypertensive, and anti-oxidative effects [10–12]. Moreover, sesamin exhibited anti-cancer effects by interfering with multiple cancer pathways [13]. A recent report suggested that sesamin can attenuate renal injury in rat models of hyperlipidemia by reducing oxidative stress and augmenting the activities of antioxidant enzymes [14]. Another study showed that sesamin exerts renoprotective effects by inhibiting neutrophil infiltration and pro-inflammatory cytokines release and enhancing adenosine-CD39-A2AR signaling [15]. Similar renoprotective effects were demonstrated against the toxicity of several xenobiotics, such as doxorubicin and fluoride [16,17]. However, sesame exhibited testicular protection in streptozotocin-induced diabetes rats [18].

The current study investigated whether sesamin can ameliorate CP’s renal and testicular toxicities by suppressing CP-induced oxidative stress, inflammation, and apoptosis. It also investigated the potential protective mechanism of sesamin.

## 2. Materials and methods

### 2.1. Sources of chemicals and analysis materials

Sesamin (>98%) was purchased from Shanghai Pureone Biotechnology (Shanghai, China) and was dissolved in carboxymethylcellulose (CMC, 0.5%). Cisplatin (1 mg/mL vial) was purchased from Sigma (St. Louis, MO) in a clinical formulation. Otherwise, all chemicals were of the highest analytical grade available commercially. The used kits in this study were predominantly purchased from Biodiagnostics Co. (Giza, Egypt), except for those used to measure serum testosterone, interleukins (IL-1 and IL-6), and tumor necrosis factor-α (TNF-α), which were brought from Abcam (Cambridge, UK), Glory Science Ltd. (Del Rio, TX), and BioSource International (Camarillo, CA), respectively.

### 2.2. Group allocation and treatment regimens

Following ethical approval from the respective body at Suez Canal University (Ismailia, Egypt) and proper animal acclimatization at the study site for one week, 32 male Wistar rats (weight: 150–180 g) were allocated into (A) normal control group: receiving only the vehicle (CMC 0.5%), (B) CP intoxicated group: receiving a single intraperitoneal dose of CP (5 mg/kg) at the 6th day of the experiment [19] plus the vehicle (CMC 0.5%), (C) CP-intoxicated group treated by sesamin 10 mg/kg: receiving CP at the dose mentioned above plus sesamin at 10 mg/kg orally for 10 days, and (D) CP-intoxicated group treated by sesamin 20 mg/kg: receiving CP plus sesamin at 20 mg/kg orally for 10 days [15].

### 2.3. Animal scarification and biochemical analyses

On the 11th experimental day, a sample of blood was withdrawn from the retro-orbital plexus in each rat (under isoflurane anesthesia), and then the rats were sacrificed by decapitation. Then, several biochemical analyses were performed on the rats’ serum (obtained after clotting and centrifugation of blood samples at 5000 rpm for 10 min) and homogenates from the rats’ renal and testicular tissues (after removing and cleaning these organs, homogenization in 0.1 mM phosphate buffer and centrifugation at 9000 r/min at 4 °C for 20 min).

The assessed biochemical parameters in the rats’ serum included urea [20], creatinine [21], IL-1, IL-6, TNF-α, and testosterone (the latter three parameters were measured according to the instructions of the kits’ providers). The assessed biochemical parameters in the renal and testicular tissue homogenates included malondialdehyde (MDA) [22], nitric oxide (NO) [23], reduced glutathione (GSH) [24], and activities of endogenous antioxidant enzymes such as glutathione peroxidase (GPx) [25], superoxide dismutase (SOD) [26], and catalase (CAT) [27].

### 2.4. Tissue preparation for histopathological and histochemical evaluation

Parts of the excised kidneys and testes were used to assess histo-morphological analysis. They were rinsed with normal saline and immediately immersed and fixed in 10% neutral buffered formalin (NBF) for 72 h. After proper tissue fixation and preservation, they were subjected to routine histological processing: dehydrated in ascending grades of ethanol (70%, 80%, 90%, and absolute alcohol), cleaned in three changes of xylene (4 min intervals), and finally implanted in paraffin wax that was melted at 60 °C. The paraffin-embedded specimens were sectioned using a rotatory microtome (YD-1508R, Zenith Lab, Pomona, CA) at 5–7 μm thickness. According to Bancroft and Gamble, the prepared paraffin sections were dewaxed and re-hydrated using the standard methods [28]. Renal and testicular tissue sections were stained with hematoxylin and eosin (H&E) to be examined by light microscopy. Further, the PAS technique detected the amount of neutral mucopolysaccharides in the sections. All photomicrographs obtained for histological and immunohistochemical evaluations were viewed and collected using an Olympus BX41 research optical microscope (Tokyo, Japan) equipped with an Olympus DP25 digital camera. The magnification scale bar was noted on the obtained photomicrographs.

### 2.5. Immunohistochemical technique

The standard streptavidin-biotin immunoperoxidase technique was carried out for cyclooxygenase-II (Cox-II) and p53 immunostaining, according to Lin and Prichard [29]. Tissue sections 5–7 μm thick were deparaffinized in three changes of xylene, hydrated in descending graded series of ethanol, and then washed with PBS (phosphate-buffered saline) and heated in citrate buffer (pH 6.0) at 120 °C in a microwave oven for 10 min to expose antigens. Endogenous peroxidase activity was blocked by incubating the section in 0.3% H_2_O_2_ for 30 min at room temperature. After that, the sections were washed in PBS and incubated with 10% goat serum for 10 min at room temperature to prevent unspecific binding. This was followed with incubation either with primary antibodies against COX-II (rabbit polyclonal COX-II specific IgG, H-62, sc-795, 50× diluted, Santa Cruz, Dallas, TX) or primary antibodies against p53 (mouse monoclonal p53 specific IgG, pab, 1801, sc-98, 100× diluted, Santa Cruz, Dallas, TX) for 120 min at 37 °C. After washing with PBS, the sections were incubated for 10 min with biotinylated secondary antibodies, followed by streptavidin–peroxidase complex for 10 min. A freshly prepared DAB was used as a chromogen for the final detection of the colorimetric reaction. Finally, the sections were counterstained with Mayer’s hematoxylin for two minutes, following the guidelines of Ramos-Vara et al. [30].

### 2.6. Data analysis

The results of biochemical serum and tissue analyses are presented as means ± standard error of mean (SEM). We employed the ANOVA followed by Tukey’s test as a post hoc analysis to evaluate the differences between study groups. The statistical significance level was set at (*p* < .05), and all analyses were done using SPSS software (version 22 for Windows, IBM, Chicago, IL).

## 3. Results

### 3.1. Biochemical serum analysis

Data analysis revealed that CP intoxication (group II) was associated with significant (*p* < .05) increases in serum urea, creatinine, IL-1, IL-6, and TNF-α concentrations, as well as a significant decrease in serum testosterone levels in comparison with normal controls. However, CP-intoxicated rats, treated with sesamin at doses of 10 mg/kg (group III) or 20 mg/kg (group IV), exhibited significantly lower (*p* < .05) serum levels of urea, creatinine, IL-1, IL-6, and TNF-α, as well as a significantly higher testosterone level in comparison with group II rats. Interestingly, treatment with sesamin at 20 mg/kg dose restored the normal concentration ranges for all assessed serum variables except for IL-6 and testosterone (Table 1).

**Table 1.** The effects of sesamin treatment on serum levels of testosterone, renal injury, and inflammatory biomarkers in cisplatin-intoxicated rats.

	Control	CP	CP + sesamin (10 mg/kg)	CP + sesamin (20 mg/kg)
Urea (mg/dL)	23.1 ± 1.15^a^	64.14 ± 2.2^b^	38.7 ± 1.6^c^	29.4 ± 1.5^a^
Creatinine (mg/dL)	0.61 ± 0.03^a^	3.14 ± 0.15^b^	1.98 ± 0.12^c^	0.76 ± 0.05^a^
IL-1 (pg/mL)	130.3 ± 8.11^a^	573.4 ± 20.3^b^	313.6 ± 12.6^c^	161.2 ± 9.9^a^
IL-6 (pg/mL)	94 ± 2.36^a^	606.5 ± 16.5^b^	316.9 ± 4.5^c^	137 ± 5.2^d^
TNF-α (pg/mL)	95.2 ± 3^a^	377.7 ± 14.4^b^	197.2 ± 10.5^c^	108.4 ± 5.9^a^
Testosterone (ng/mL)	0.83 ± 0.04^a^	0.22 ± 0.01^b^	0.41 ± 0.02^c^	0.72 ± 0.02^d^

CP: cis-diamminedichloroplatinum/cisplatin; IL: interleukin; TNF-α: tumor necrosis factor-α.

Data are presented as means ± SEM (*n* = 8). Means within the same row carrying different superscripts are significantly different at (*p* < .05).

### 3.2. Biochemical renal tissue analysis

Cisplatin-intoxicated rats showed significant increases in renal tissue levels of MDA and NO, as well as a significant reduction in renal tissue GSH concentration and activities of antioxidant enzymes (GSH, SOD, and CAT), compared to normal control rats. On the contrary, treatment of CP intoxication with either dose of sesamin (10 or 20 mg/kg) significantly ameliorated all these alterations in a dose-dependent manner; the normal tissue concentration ranges were restored only in the group treated with sesamin at 20 mg/kg dose (Table 2).

**Table 2.** The effects of sesamin treatment on renal tissue lipid peroxidation and antioxidant compounds in cisplatin-intoxicated rats.

	Control	CP	CP + sesamin (10 mg/kg)	CP + sesamin (20 mg/kg)
MDA (nmol/g)	81.12 ± 2.9^a^	154.4 ± 5.3^b^	116.7 ± 3.9^c^	93.13 ± 2.06^a^
NO (μmol/g)	56.2 ± 1.4^a^	101 ± 2.7^b^	72.6 ± 2.8^c^	60.6 ± 2.6^a^
GSH (μmol/g)	7.9 ± 0.19^a^	3.5 ± 0.22^b^	5.5 ± 0.16^c^	7.4 ± 0.18^a^
GPx (U/g)	37.2 ± 1.12^a^	18.2 ± 1.34^b^	26.6 ± 0.98^c^	34.12 ± 1.1^a^
SOD (U/g)	62.6 ± 2.6^a^	31.9 ± 1.2^b^	48.7 ± 2.3^c^	58 ± 2.25^a^
CAT (U/g)	5.2 ± 0.25^a^	2.7 ± 0.07^b^	3.7 ± 0.13^c^	4.6 ± 0.13^a^

CAT: catalase; CP: cis-diammine-dichloroplatinum/cisplatin; GPx: glutathione peroxidase; GSH: glutathione; MDA: malondialdehyde; NO: nitric oxide; SOD: superoxide dismutase.

Data are presented as means ± SEM (*n* = 8). Means within the same row carrying different superscripts are significantly different at (*p* < .05).

### 3.3. Biochemical testicular tissue analysis

Cisplatin intoxication in group II rats was associated with significant elevations in testicular tissue MDA and NO concentrations with simultaneous reductions in GSH concentration and antioxidant enzymatic activities compared with control rats. Treatment of CP intoxication with sesamin at either dose (10 or 20 mg/kg) ameliorated all these alterations; however, the testicular tissue GSH concentration was not significantly increased with the 10 mg/kg dose compared to CP-intoxicated rats. Rats in group IV showed comparable levels of all assessed tissue parameters to the control group, except for testicular tissue NO and SOD (Table 3).

**Table 3.** The effects of sesamin treatment on testicular tissue lipid peroxidation and antioxidant compounds in cisplatin-intoxicated rats.

	Control	CP	CP + sesamin (10 mg/kg)	CP + sesamin (20 mg/kg)
MDA (nmol/g)	203.6 ± 7.5^a^	585.2 ± 20.1^b^	295.4 ± 8.8^c^	233.8 ± 5.2^a^
NO (μmol/g)	77.1 ± 2.1^a^	171.4 ± 3.66^b^	122 ± 2.4^c^	90.3 ± 3.8^d^
GSH (μmol/g)	6.6 ± 0.23^a^	4.85 ± 0.36^b^	5.48 ± 0.3^ab^	6.2 ± 0.22^a^
GPx (U/g)	10.04 ± 0.24^a^	4.01 ± 0.31^b^	6.3 ± 0.2^c^	9.3 ± 0.32^a^
SOD (U/g)	75.4 ± 2.9^a^	35.6 ± 1.1^b^	51.5 ± 1.7^c^	65.6 ± 2.4^d^
CAT (U/g)	21.6 ± 1.2^a^	10.8 ± 0.54^b^	15.6 ± 0.5^c^	19.7 ± 1.1^a^

CAT: catalase; CP: cis-diammine-dichloroplatinum/cisplatin; GPx: glutathione peroxidase; GSH: glutathione; MDA: malondialdehyde; NO: nitric oxide; SOD: superoxide dismutase.

Data are presented as means ± SEM (*n* = 8). Means within the same row carrying different superscripts are significantly different at (*p* < .05).

### 3.4. Histopathological findings

**Figure 1.**
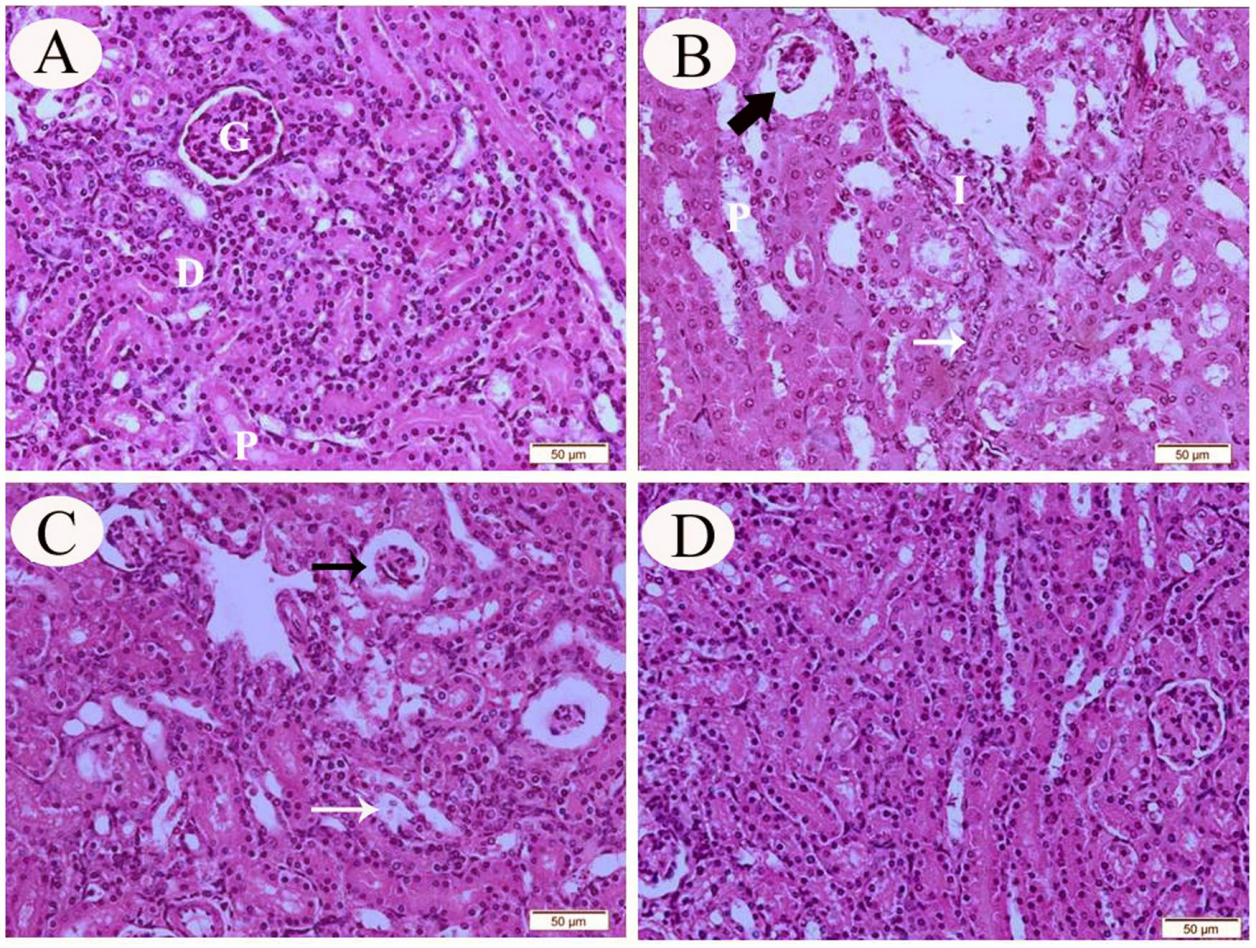
Representative photomicrograph (H&E stain) of renal tissue sections from control (A), CP-intoxicated (B), group III (CP-SES 10 mg/kg) (C), and group IV (CP-SES 20 mg/kg) rats (D). Renal glomeruli (G), proximal tubules (P), and distal tubules (D). Sections, obtained from CP-intoxicated rats, showed patchy mononuclear inflammatory infiltrate (I) and tubular injury with cellular atypia in the proximal tubules (P). Some of these tubulocytes revealed cytoplasmic vacuolization and focal apoptosis (white thin arrow). The glomeruli and the blood capillaries were atrophied and reduced in size (black thick arrow). Renal sections from group III rats showed minimal histopathological findings with less pronounced tubular degenerative changes (white thin arrow) and shrunken renal glomeruli (black thin arrow). Renal sections from group IV rats revealed an improvement and unity in renal tissue with no histological alterations.

The kidneys of control rats exhibited normal histological findings along with normal tubular, corpuscular, and interstitial architecture (Figure 1(A)). Meanwhile, kidneys obtained from CP-intoxicated rats showed histopathological abnormalities in the renal interstitia, glomeruli, and tubular epithelia. The peritubular interstitium showed patchy mononuclear inflammatory cell infiltrates, while renal proximal tubular cells (tubulocytes) showed atypia with frequent cytoplasmic vacuolization and focal apoptosis. The glomeruli were atrophied and reduced in size when compared to the control group (Figure 1(B)). The kidneys of group III (CP + sesamin 10 mg/kg) rats showed minimal histopathological alterations with less pronounced tubular degenerative changes and shrunken renal glomeruli (Figure 1(C)). Renal sections from group IV (CP + sesamin 20 mg/kg) revealed near-normal renal architecture with no histopathological alterations (Figure 1(D)).

**Figure 2.**
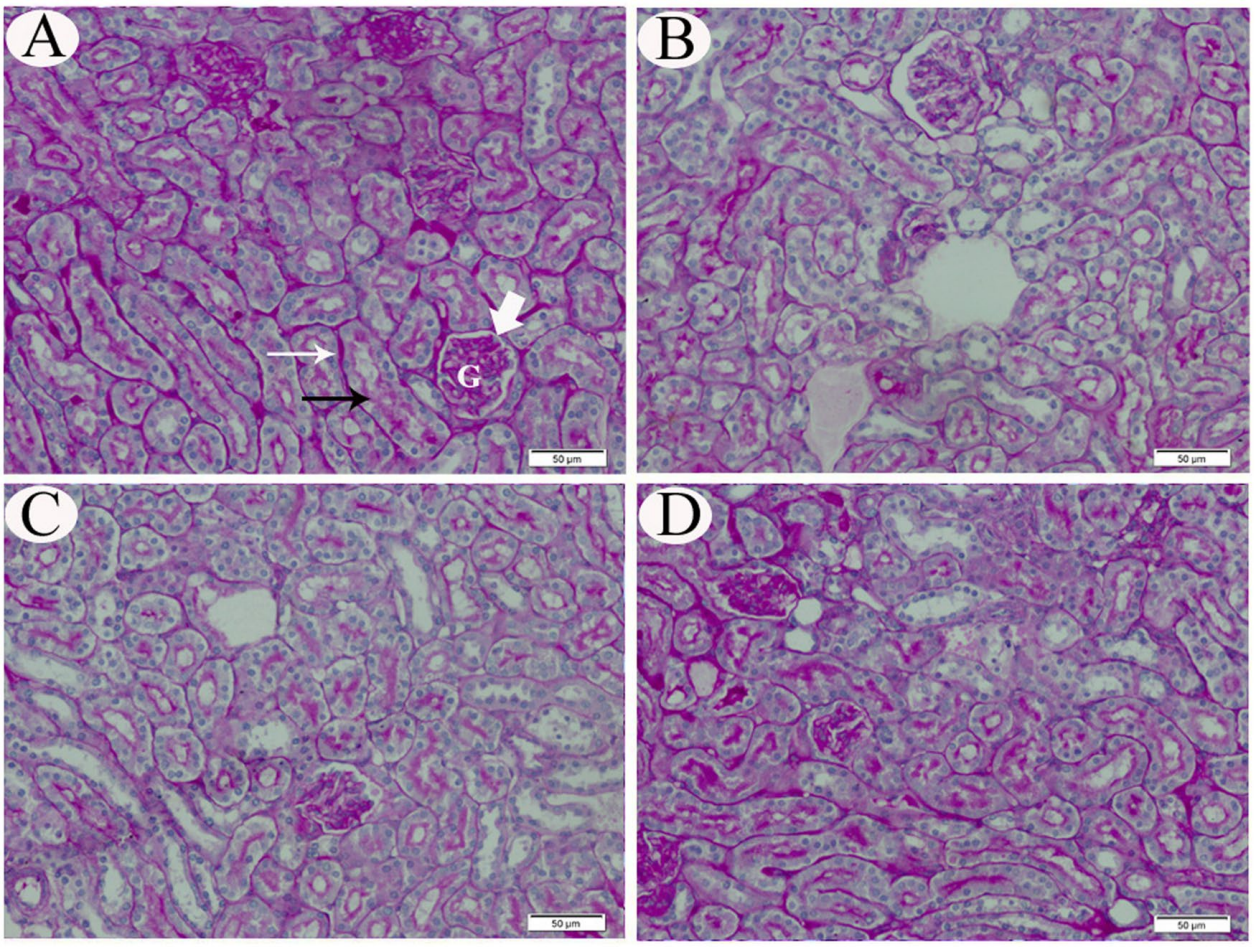
Representative photomicrograph (PAS stain) of renal tissue sections from control (A), CP-intoxicated (B), group III (CP-SES 10 mg/kg) (C), and group IV (CP-SES 20 mg/kg) rats (D). Cortical tissues of control rats (double walls of Bowman’s capsule (white thick arrow), capillaries of the glomeruli (G), tubular basement membranes (white thin arrow), and the brush borders of the proximal tubules (black thin arrow)) exhibited strong PAS-positive reaction (A). The tubular cytoplasm was stained faintly, while the nuclei showed PAS negative reaction. Renal sections from rats, exposed to CP only, demonstrated a severe reduction of mucopolysaccharide contents in comparison to the renal tissue of control rats. Microscopic observation of group III rats’ kidneys revealed marked diminution in PAS-positive material in the renal tissues, while the kidneys of group IV rats exhibited normal PAS reaction in the glomeruli, Bowman’s capsules, the tubular basement membranes, and the brush borders of the proximal tubules.

The neutral mucopolysaccharide content of the renal tissues was demonstrated by PAS staining as magenta-stained regions. Cortical tissues of control rats (double walls of Bowman’s capsule, capillaries of the glomeruli, tubular basement membranes, and the brush borders of the proximal tubules) exhibited PAS-positive solid reactions. The tubular cytoplasm was stained faintly, while the nuclei showed PAS-negative reaction (Figure 2(A)). Examination of renal tissue sections from rats exposed to CP only demonstrated a severe reduction of polysaccharides content in comparison to the renal tissues from control rats (Figure 2(B)). Microscopic examination of group III rats’ kidneys exhibited marked diminution in PAS-positive material (Figure 2(C)). In contrast, the kidneys of group IV rats revealed a normal PAS reaction in the glomeruli, Bowman’s capsules, the tubular basement membranes and the brush borders of the proximal tubules (Figure 2(D)).

**Figure 3.**
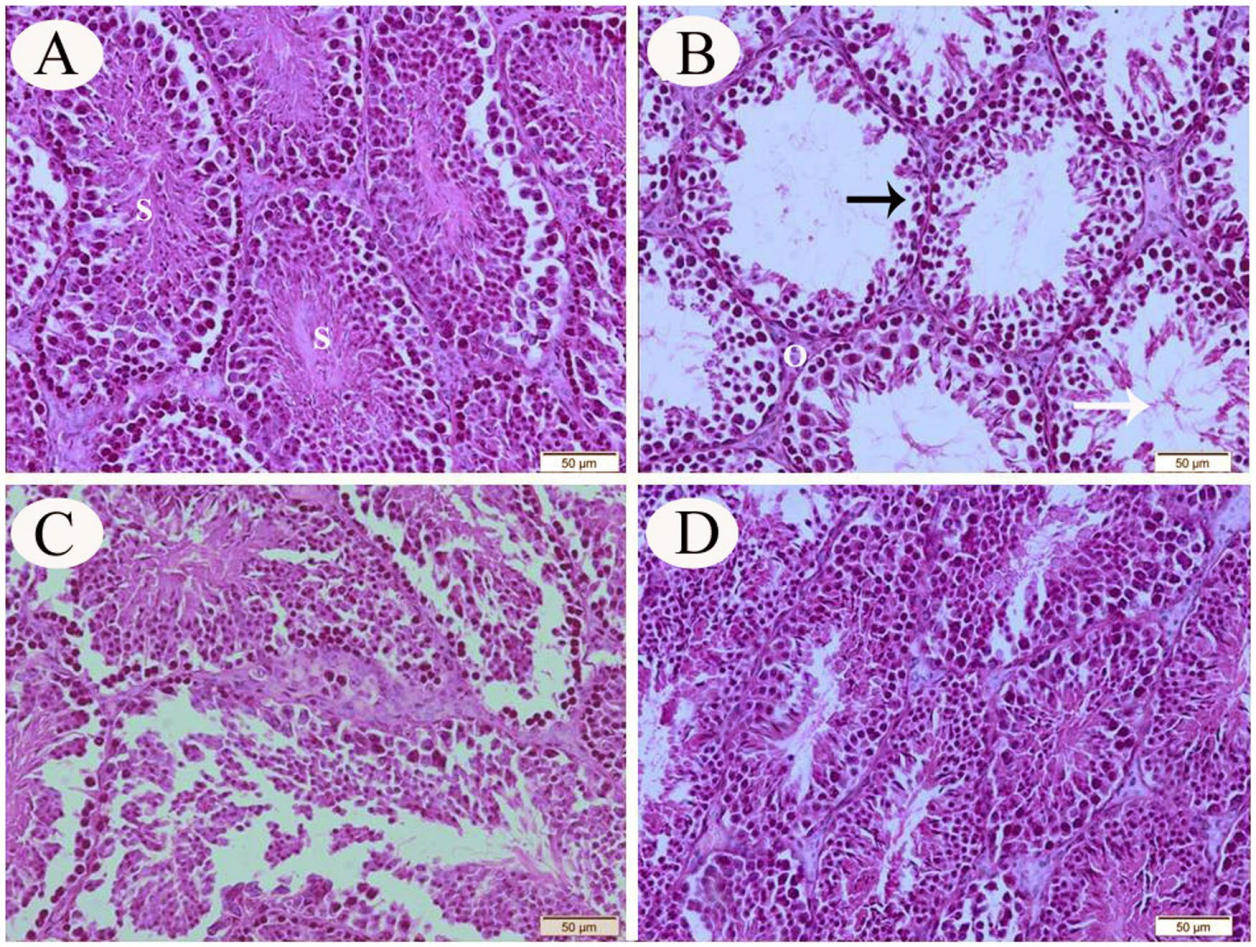
Representative photomicrograph (H&E stain) of testicular tissue sections from control (A), CP-intoxicated (B), group III (CP-SES 10 mg/kg) (C), and group IV (CP-SES 20 mg/kg) rats (D). The testicular sections from control rats appeared normal with typical spermatogenic cell morphologies (S). On the other hand, testicular sections from CP-intoxicated rats revealed obvious histological alterations; an accentuated cellular depletion, and degenerative alterations in the seminiferous epithelium (black arrow). In addition, significant reductions in the tubular diameter and seminiferous germinal epithelium height were noted when compared with control rats. Cellular debris within the tubular lumen (white arrow) and interstitial edema were seen (O). The testicular histopathological alterations that appeared in group III were less prominent than those recorded in CP-intoxicated rats. Normal spermatogenic cell series of the seminiferous epithelium were observed in the testicular sections of group IV rats.

In cross-sections prepared from seminiferous tissue specimens obtained from control rats, the histological testicular architecture appeared normal with regular spermatogenic cell morphology (Figure 3(A)). On the contrary, testicular sections from the CP-intoxicated animals revealed an accentuated cellular depletion and degenerative alterations in the seminiferous epithelium, cellular debris within the tubular lumen and interstitial edema, as well as a significant reduction in the tubular diameter and seminiferous germinal epithelium height when compared with control rats (Figure 3(B)). The testicular histopathological alterations were less prominent in group III than those recorded in the CP-only exposed group (Figure 3(C)). Normal spermatogenic cell series of the seminiferous epithelium were observed in the testicular sections of group IV rats (Figure 3(D)).

**Figure 4.**
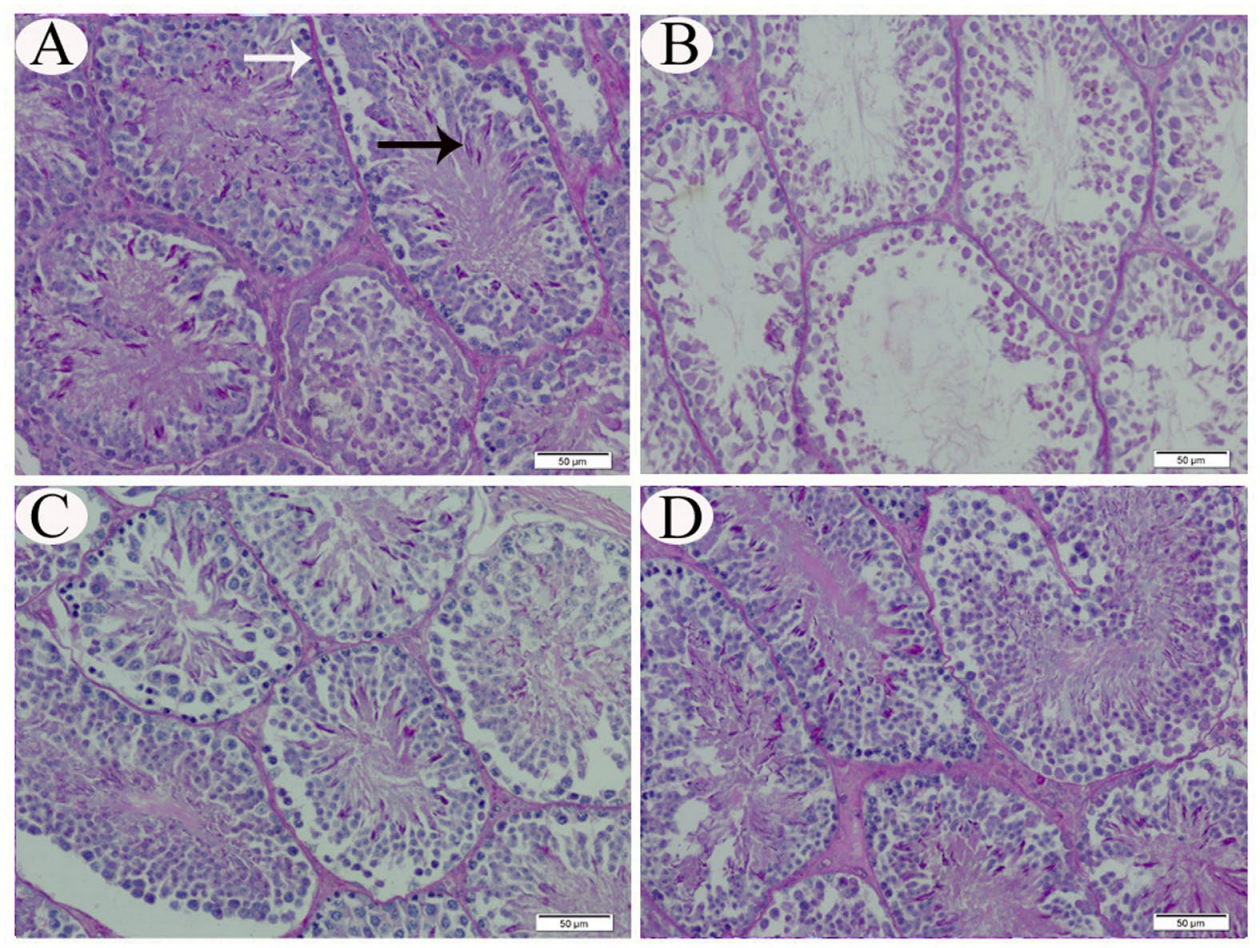
Representative photomicrograph (PAS stain) of testicular tissue sections from control (A), CP-intoxicated (B), group III (CP-SES 10 mg/kg) (C), and group IV (CP-SES 20 mg/kg) rats (D). PAS-positive particles were observed in the cytoplasm of upper series cells of the germinal epithelium close to the lumen of seminiferous tubules (black arrow) and the tubular basement membrane (white arrow) of the testicular tissue sections from control and group IV rats. In contrast, PAS-positive materials were detected less in CP-intoxicated and group III rats.

Regarding mucopolysaccharides contents of the testicular tissues, PAS-positive particles were observed in the cytoplasm of upper series cells of the germinal epithelium, close to the lumen of seminiferous tubules, as well as the tubular basement membrane of testicular tissue sections of control and group IV rats (Figure 4(A,D)). In contrast, PAS-positive materials were detected less in CP-only treated and group III rats (Figure 4(B,C)).

### 3.5. Immunohistochemical evaluation

**Figure 5.**
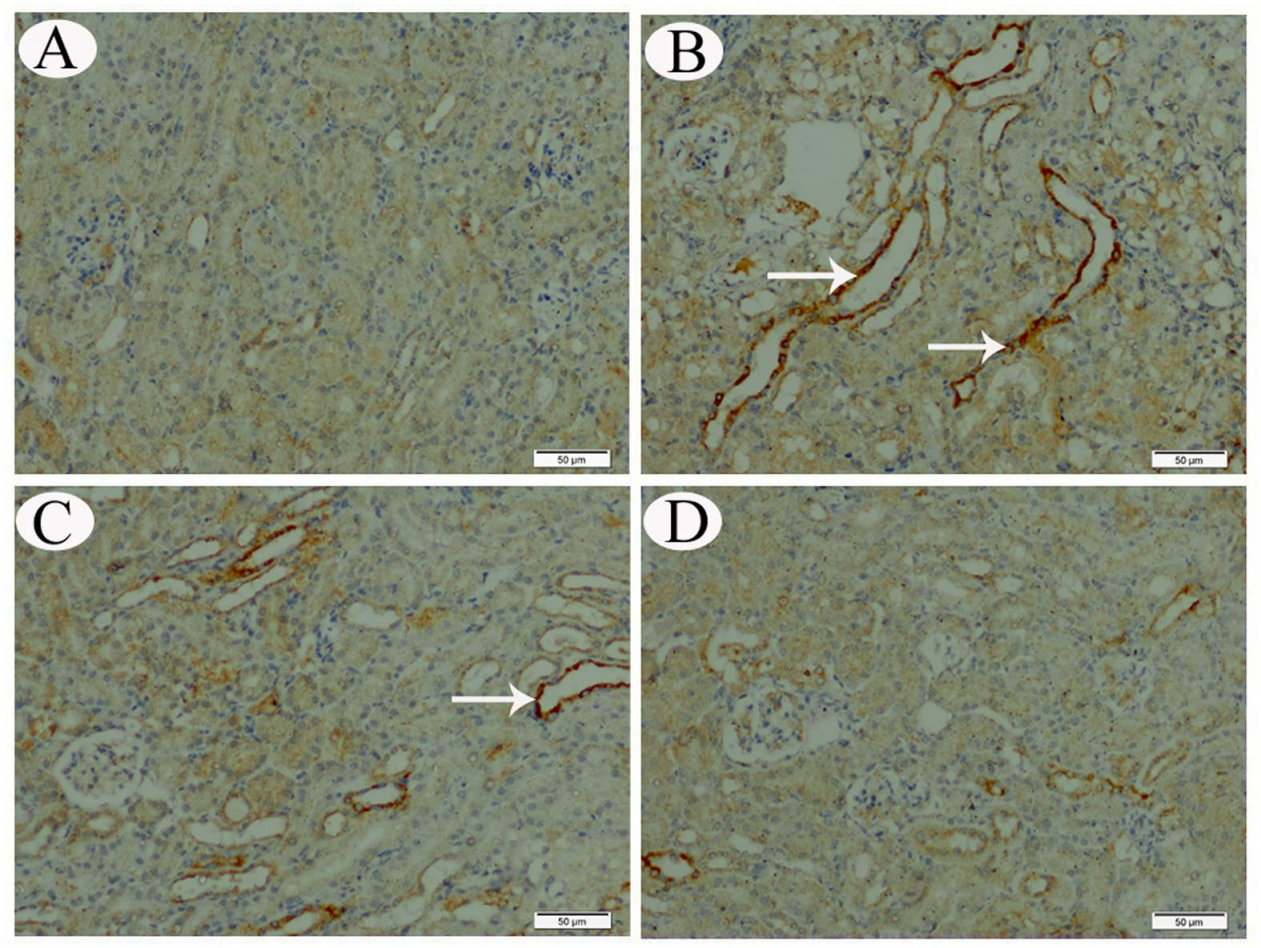
COX-II IHC expression in renal tissue sections from control (A), CP-intoxicated (B), group III (CP-SES 10 mg/kg) (C), and group IV (CP-SES 20 mg/kg) rats (D). No COX-II immunoreaction was noted in the renal tissue sections from control rats. Meanwhile, marked COX-II-positive immunostaining was observed in the sections obtained from CP-intoxicated and group III rats (white arrows). Occasionally, the cortical tissue sections of rats in group IV displayed low COX-II reactivity (white arrows).

**Figure 6.**
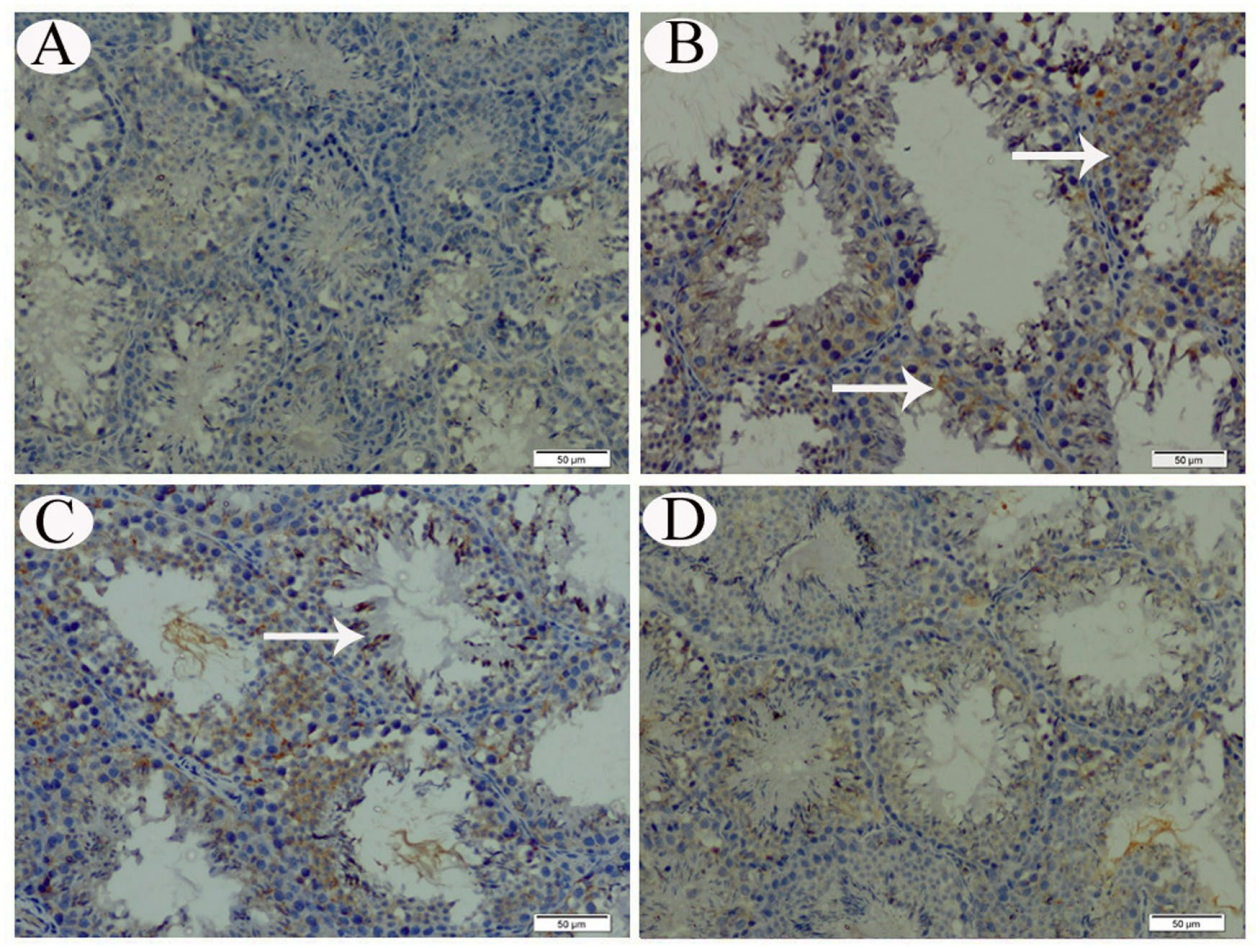
COX-II IHC expression in testicular tissue sections from control (A), CP-intoxicated (B), group III (CP-SES 10 mg/kg) (C), and group IV (CP-SES 20 mg/kg) rats (D). No COX-II immunoreaction was noted in the testicular tissue of control rats. Meanwhile, a marked COX-II-positive immunostaining was observed in sections, obtained from CP-intoxicated and group III rats (white arrows). Occasionally, the testicular tissue sections from rats in group IV displayed low COX-II reactivity (white arrows).

**Figure 7.**
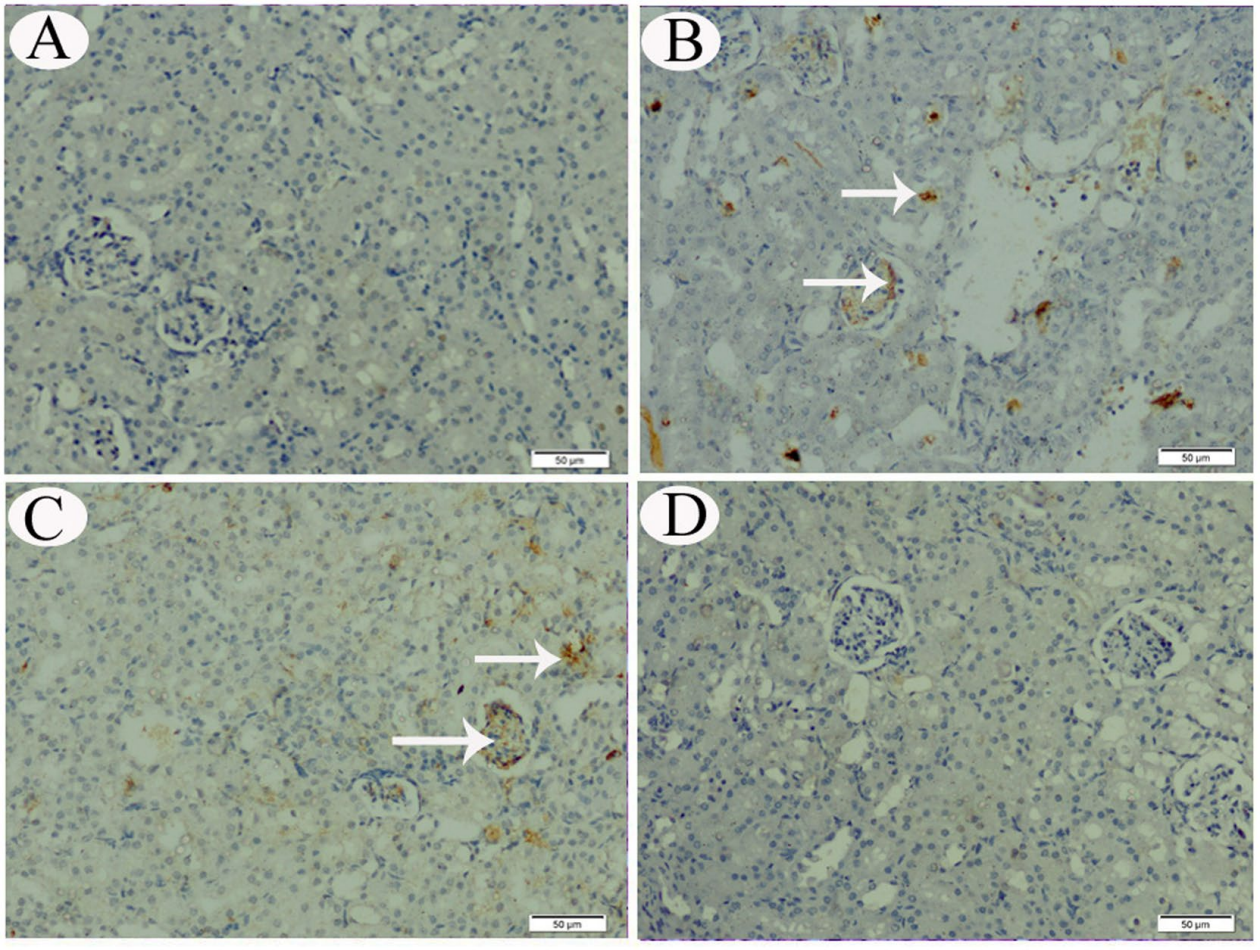
p53 IHC expression in renal tissue sections from control (A), CP-intoxicated (B), group III (CP-SES 10 mg/kg) (C), and group IV (CP-SES 20 mg/kg) rats (D). p53 immunoreaction was marked in the renal tissue sections of CP-intoxicated and group III rats (white arrows).

**Figure 8.**
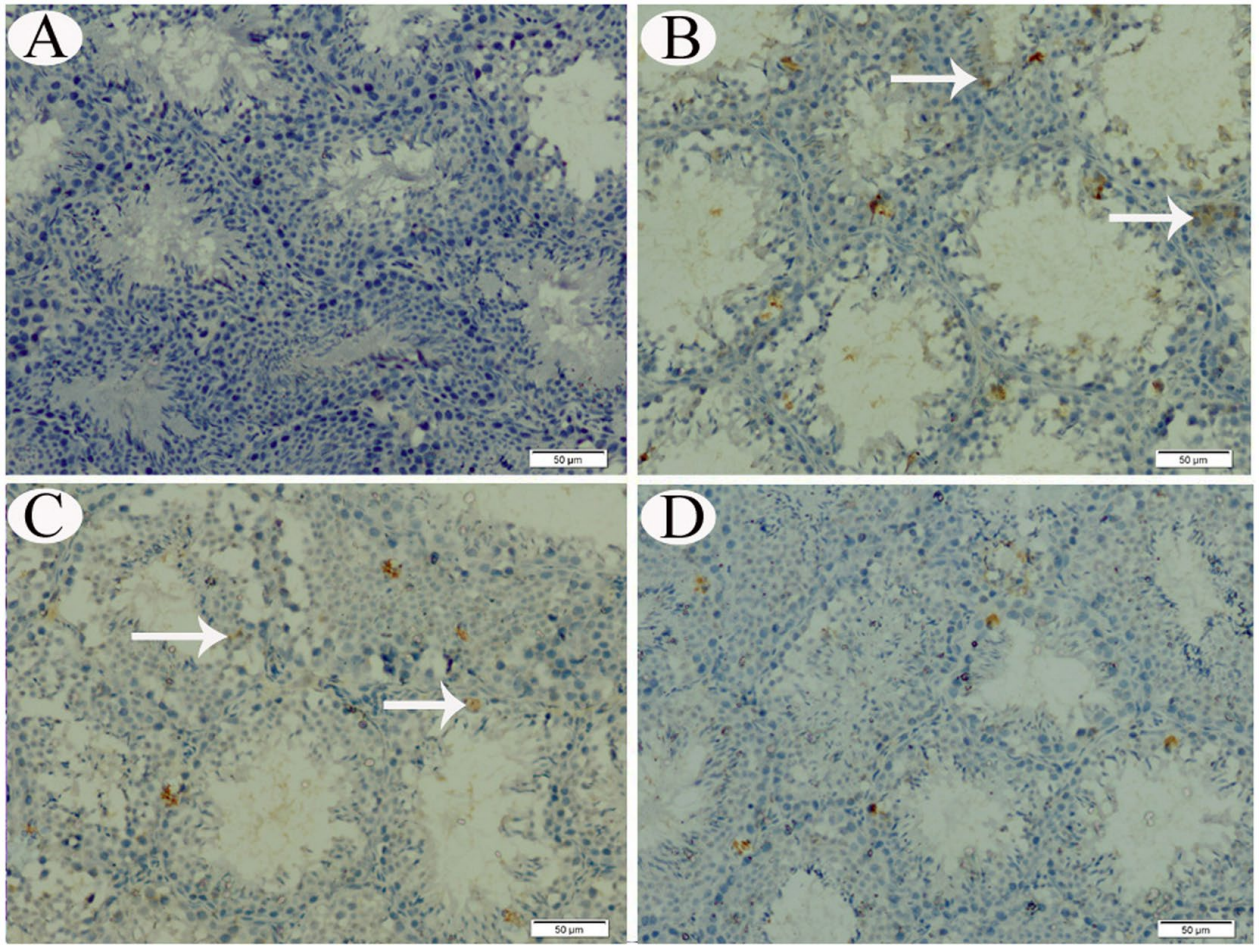
p53 IHC expression in testicular tissue sections from control (A), CP-intoxicated (B), group III (CP-SES 10 mg/kg) (C), and group IV (CP-SES 20 mg/kg) rats (D). p53 immunoreaction was marked in the testicular tissue sections of CP-intoxicated and group III rats (white arrows).

No COX-II immunoreaction was observed in control animals’ renal and testicular tissues (Figures 5(A) and 6(A)). Meanwhile, distinctly high levels of COX-II positive immunostaining were observed in the obtained sections from CP-treated and group III animals (Figures 5(B,C) and 6(B,C)). The animals’ cortical renal and testicular tissue sections in group IV occasionally displayed low COX-II reactivity (Figures 5(D) and 6(D)). Similarly, no p53 immunoreaction was observed in control animals’ renal and testicular tissues (Figures 7(A) and 8(A)). Immunohistochemical analysis revealed that p53 immunoreaction was significantly upregulated in the renal and the testicular tissue sections of CP-treated animals and group III (Figures 7(B,C) and 8(B,C)). Renal and testicular tissue sections from group IV animals significantly ameliorated p53 expression compared to CP-only exposed rats (Figures 7(D) and 8(D)).

## 4. Discussion

Our study highlights the protective effects of sesamin against CP-induced renal and testicular toxicities. Compared to normal controls, CP-intoxicated rats showed significant deterioration of renal and endocrine testicular functions, as well as abnormal histological architecture. However, adding sesamin reversed most toxic effects of CP. It significantly reduced serum levels of pro-inflammatory cytokines (IL-1, IL-6, and TNF-α), inflammatory cell infiltrates on HP examination, and renal and testicular tissue COX-II enzyme expression. Moreover, it enhanced the activities of endogenous antioxidant enzymes (GSH, SOD, and CAT) and reduced tissue MDA and NO concentrations in renal and testicular tissues [19]. Further, HP and IHC examinations revealed fewer foci of apoptosis and lower expression of p53 in sesamin-treated rats. Thus, the protective effects of sesamin observed in this study probably occur via antioxidant, anti-inflammatory, and anti-apoptotic mechanisms.

Several studies have explored the mechanisms behind CP-induced toxicity. After entering tubular cells, CP undergoes a series of reactions that lead to the formation of more toxic metabolites [2]. It induces oxidative stress through tipping the balance between free radical production and elimination. Following previous findings [19,31,32], CP-intoxicated rats exhibited significant reductions in the activities of antioxidant enzymes (GSH, SOD, and CAT), as well as increased tissue levels of MDA and NO. Further, CP interacts with GSH inside the cells, causing its depletion and interfering with the mitochondrial respiratory chain, producing reactive oxygen species [33].

In our study, CP treatment was associated with increased serum levels of several pro-inflammatory cytokines, such as ILs and TNF-α. The latter can directly induce cytotoxicity in glomerular and epithelial cells of the renal tubules. Further, Zhang et al. demonstrated that upregulating TNF-α is crucial in CP-mediated inflammation. Using mice with toll-like receptor-4 (TLR4) knock-out, they showed that the CP-induced increase in TNF-α expression was mainly TLR4-dependent [34]. The increased IL-1 levels in CP-intoxicated rats contribute to prostaglandin E2 production in mesangial cells, changing the hemodynamics of the glomeruli. In contrast, the increased IL-6 levels stimulate the production of fibronectin, increasing the thickness of the glomerular basement membrane [35,36].

Histopathological analysis of renal and testicular tissues showed atypic injuries and degenerative changes in renal and seminiferous tubules. This confirms the previous findings on CP toxicity in the literature [19,37,38]. The low number of PAS-positive cells in the renal and testicular tissue indicates depletion of the neutral mucopolysaccharides content [39,40]. This may be caused by initiation of necrosis, lipid peroxidation, and increased catabolic rates [41,42].

Regarding the results of the IHC examination, our study extends the evidence on the involvement of COX-II in CP toxicity [43,44]. COX-II produces pro-inflammatory prostaglandins and thromboxanes, reducing renal blood flow [45]. Former investigators have shown that inhibiting COX-II expression can alleviate CP toxicity [43,44]. In parallel, recent studies have demonstrated that p53 plays a crucial role in CP-induced cellular apoptosis through regulating autophagy and MAPK pathways [46,47]. These conclusions agree with our research because p53 immunoreaction was significantly more intense in rats treated with CP.

On the other hand, the results of this study illustrate how sesamin can antagonize the toxic actions of CP in both renal and testicular tissues. First, sesamin exerted potent antioxidant effects in CP-intoxicated rats, as manifested by the significant increases in tissue GSH concentration and activities of antioxidant enzymes. These results add to the published evidence on the antioxidant activity of sesamin [13,16]. Notably, Hsu et al. studied the effect of sesame oil (which contains sesamin) on CP-induced hepatorenal toxicities. They concluded that sesame oil blocks CP-induced lipid peroxidation, hydroxyl radical, and peroxynitrite production in renal and hepatic tissues. Interestingly, sesame oil supplementation did not interfere with the anti-tumor effects of CP [48]. Along with the anti-proliferative effects of sesamin shown in other studies [12,49], it is unlikely to hinder the tumor-suppressing activity of CP.

The anti-inflammatory effects of sesamin are proven in several findings in our study, such as reduction of serum concentrations of pro-inflammatory cytokines, amelioration of inflammatory cell infiltration on HP examination, and down-regulated expression of COX-II enzyme on IHC examination. In a mouse model of renal ischemia-reperfusion injury, sesamin administration decreased serum levels of renal injury markers and tissue neutrophil infiltrates, IL-1, and TNF-α levels [14]. In another study, sesamin supplementation ameliorated the CCL-4-induced increases in hepatic tissue IL-1 and COX-II expression [11]. Similarly, attenuation of tissue inflammation on HP examination in sesamin-treated animals was previously demonstrated [11,16]. Along with our results, these findings confirm the anti-inflammatory effects of sesamin.

The reduced degree of apoptosis on HP examination of sesamin-treated rats’ tissues may be explained by the reduction of p53 expression on IHC examination in the same group. Similar results have been reported for sesamin in rats with reversible middle cerebral artery occlusion [50]. However, Siao et al. reported that sesamin increases the expression of p53 in breast cancer MCF-7 cells [51], which may indicate the differential roles of sesamin in cancer and normal tissue cells. Cao et al. have shown different anti-apoptotic mechanisms for sesamin, such as inhibiting the activities of caspase-3, p-JNK, and Bax proteins, as well as suppressing the release of mitochondrial cytochrome c into the cytoplasm [17].

Regarding sesamin dosage administration, the 20 mg/kg dose had more significant effects on almost all tested parameters than the 10 mg/kg dose. The higher dose could restore the expected levels of most tested parameters and prevent CP-induced abnormalities in tissue architecture. Such dose-dependent effects have been noted previously for sesamin [9,15]. The observed discrepancy between the effects of these doses highlights the value of dose optimization in future studies and possible therapeutic applications.

The nephroprotective potential of sesamin against other anti-cancer drugs and xenobiotics was previously examined. Guo et al. found that treatment with sesamin could attenuate doxorubicin-induced hepatorenal toxicities by enhancing antioxidant enzyme activity [16]. Other studies showed the cytoprotective effects of sesamin against fluoride [16], alcohol [52], carbon tetrachloride [11,52], hyperglycemia [53,54], and hyperlipidemia [13]. These results open the door for investigating the chemopreventive value of sesamin against a wide range of biological and chemical cell injuries [15]. Future studies should also compare the cytoprotective efficacy of sesamin to other established antioxidant compounds and test their efficacy in combination. Further, an improved understanding of the underlying mechanisms of CP renal and testicular toxicities is needed.

In conclusion, sesamin could protect the kidneys and testes against CP toxicity through its antioxidant, anti-inflammatory, and anti-apoptotic effects. Further experimental and clinical studies are warranted to determine the future therapeutic applications of these findings.

## Data Availability

Data are available from the corresponding author upon reasonable request.
